# Takotsubo syndrome induced by brachytherapy in a patient with endocervical adenocarcinoma

**DOI:** 10.1186/s40959-020-00082-8

**Published:** 2020-12-05

**Authors:** Aline Cristini Vieira, Mauricio Fernando Silva Almeida Ribeiro, Julianne Lima, Jacob Sessim Filho, Heloisa de Andrade Carvalho, Max Senna Mano

**Affiliations:** 1 Oncology Center, Hospital Sírio Libanês, Rua Dona Adma Jafet, 91. 2nd floor. Building A, 01308-050 São Paulo, Brazil; 2 Cardiology Center, Hospital Sírio-Libanês, São Paulo, Brazil; 3 Department of Radiation Oncology, Hospital Sírio-Libanês, São Paulo, Brazil; 4 Hospital das Clinicas HCFMUSP, Faculdade de Medicina, Universidade de Sao Paulo, Sao Paulo, Brazil

**Keywords:** Takotsubo, Brachytherapy, Endocervical adenocarcinoma, Heart failure, Stress cardiomyopathy, Broken heart syndrome

## Abstract

**Background:**

Takotsubo syndrome (TTS), also known as stress cardiomyopathy, apical ballooning syndrome and broken heart syndrome, is characterized by acute-onset chest pain, electrocardiographic (ECG) abnormalities and reversible left ventricular (LV) disfunction in the absence of a culprit obstructive lesion in the coronary arteries; therefore, myocardial infarction is the most important differential diagnosis. Usually induced by emotional/physical stress, its treatment consists in hemodynamic support until complete and spontaneous recovery occurs, which is generally achieved within a few days to weeks. Cervical malignancies are an important public health issue in low/middle-income countries and, in the setting of locally advanced disease, concurrent chemoradiation followed by brachytherapy is considered the standard treatment, harboring curative potential.

**Case report:**

We report a case of a 38-year-old woman who underwent concurrent chemoradiotherapy and developed cardiopulmonary arrest in ventricular fibrillation during a brachytherapy session. Complementary tests disclosed altered ECG and cardiac biomarkers, no evidence of coronary artery obstruction, as well as LV disfunction consistent with TTS on echocardiogram and cardiac MRI. After few days of supportive therapy, complete recovery of heart function was observed.

**Conclusions:**

Especially for cancer patients, who usually experience intense emotional/physical stress intrinsically associated with their diagnosis and aggressive treatments, considering TTS as a differential diagnosis is warranted. Intracavitary brachytherapy procedure may represent a trigger for TTS.

## Introduction

Takotsubo syndrome (TTS), also known as stress cardiomyopathy, apical ballooning syndrome and broken heart syndrome, is an acute and transient left ventricular (LV) myocardial dysfunction, which can occur in the setting of a severe psychological or physical stress event, most often occurring 1 to 5 days before [1]. TTS’s clinical presentation might be indistinguishable from an acute coronary syndrome (ACS) with respect to symptoms, electrocardiographic (ECG) changes and biomarkers. Since its first report in 1990 by Sato et al. [2]. TTS remains with no reliable non-invasive diagnostic approach, leaving coronary angiography with left ventriculography as the gold standard diagnostic tool to reject or to ratify this diagnosis to this days [3, 4]. Typically, it occurs in postmenopausal women with few cardiovascular risk factors [1, 3–5].

The exact pathophysiology of TTS is still unknown [6]. Besides anecdotal case reports describing the occurrence of TTS in the setting of malignancy and chemotherapy, the role of chemotherapy and tumor in the development of TTS with regard to physical and emotional stress remains unclear [7]. It has been suggested that the treatment of the malignancy itself is associated with the development of TTS [8].

Herein we describe a case of a 38-year-old female patient without history of cardiovascular disorder who developed TTS during a brachytherapy session to treat an endocervical adenocarcinoma.

## Case report

A 38-year-old female presented with an abnormal pap smear in January 2018 without local or systemic manifestations. A biopsy was performed and the patient was diagnosed with endocervical adenocarcinoma. A staging magnetic resonance imaging (MRI) of the pelvis and a positron emission tomography – computed tomography (PET-CT) were performed, which showed a hypermetabolic bulky lesion arising from uterine cervix (SUV max: 23.0), without parametrial, bladder, rectal involvement, or distant metastasis. Clinical staging based on International Federation of Gynecology and Obstetrics (FIGO) classified the disease in FIGO IIA1.

The patient started chemoradiotherapy with cisplatin 40 mg/m^2^ weekly, and high dose-rate brachytherapy starting in the fourth week of external beam irradiation. At the end of the third session (four brachytherapy sessions programmed), during applicator removal, although under anesthesia, the patient developed a ventricular tachycardia with pulse, followed by cardiopulmonary arrest in ventricular fibrillation. After two minutes of cardiopulmonary resuscitation, return of spontaneous circulation was achieved, and the patient was admitted to an intensive care unit.

**Fig. 1:**
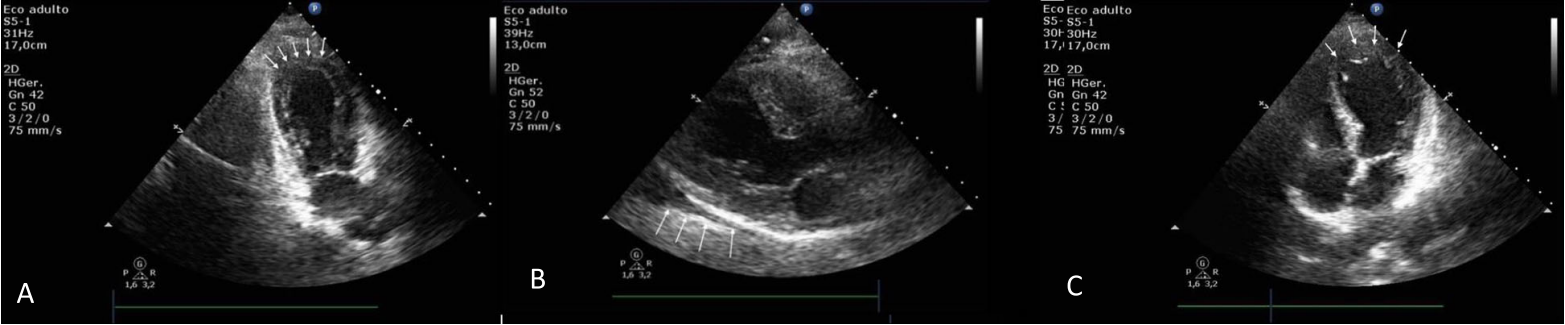
Apical 4 chambers view showing akinesia of the mid-apical segments of the inferior septal and anterolateral walls, determining apical ballooning (arrows). LA: Left Atrial. LV: Left Ventricle. RA: Right Atrial. RV: Right Ventricle

**Fig. 2:**
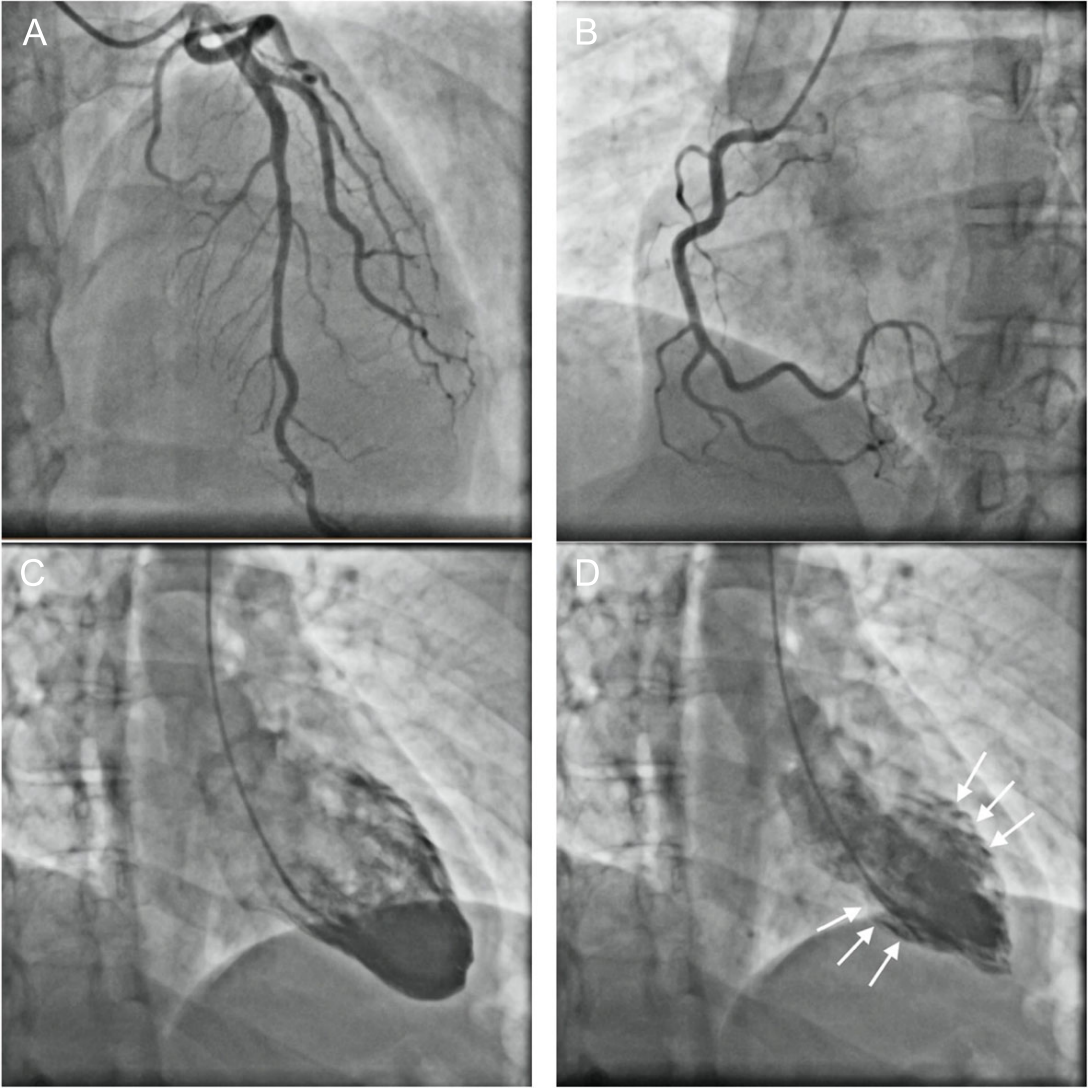
Cineangiocoronariography showing absent of significant obstructive atherosclerotic lesions. Left coronary in right anterior cranial oblique incidence (**a**). Right coronary in left anterior cranial oblique incidence (**b**). Cardiac ventriculography demonstrating mid left ventricular segments hypokinesia (arrows). Left ventricle in max diastole (**c**) and in max systole (**d**) in right oblique incidence.

**Fig. 3:**
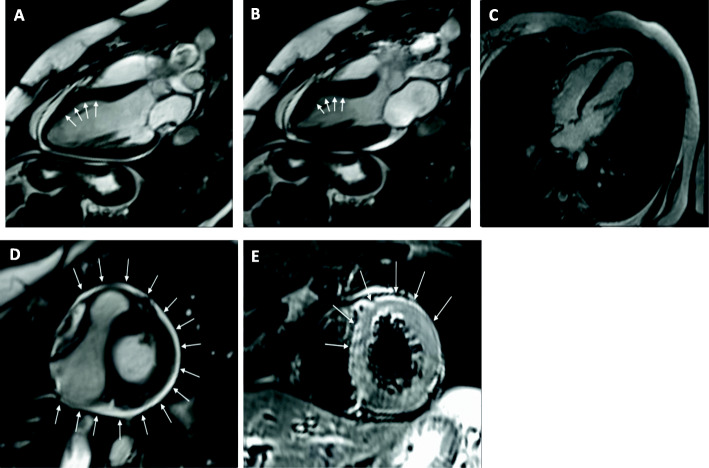
Cine cardiac magnetic resonance images showing a 3-chamber view of the left ventricle in end diastole (**a**) and in end systole (**b**) demonstrating a circumferential mid-ventricle hypokinesia with greater intensity in the anteroseptal segment (arrows). (**c**) image shows late gadolinium enhancement sequence showing a 4-chamber view of the heart without any sign of myocardial fibrosis. (**d**) Cine cardiac magnetic resonance sequence demonstrating diffuse pericardium effusion of moderate intensity (arrows). (**e**) Short-tau inversion-recovery (STIR) sequence, a T2-weighted sequence used to assess myocardial edema, demonstrating the presence of hypersignal in the middle segments of the anterior, septal and lateral walls (arrows) which indicates the presence of myocardial edema in these segments

During the investigation, ECG showed ST-segment elevation in lead II and in AVF, and prolonged QT interval of 470 msec. There was a rise in the levels of serum creatine kinase-MB (CKMB) level to 10.4 ng/mL (normal limit: < 5.0 ng/mL), serum troponin-I level was 2.04 ng/ml (normal limit: < 0.16 ng/ml) and natriuretic peptide B levels (BNP) to 233 pg/mL (normal limit: < 100 pg/mL). Transthoracic echocardiogram showed marked LV dysfunction, with akinesia of all apical and middle segments of LV wall, with estimated LV ejection fraction (LVEF) of 30% (Fig. 1). The coronary angiography demonstrated no obstructive lesions in the coronary arteries and moderate hypokinesia of the anterior and inferior apical segments of LV wall (Fig. 2). Holter showed rare and isolated polymorphic ventricular premature beats and signs of heart dysautonomia with parasympathetic depression and adrenergic predominance. A cardiac MRI was performed seven days after the event showing circumferential mid-ventricle hypokinesia associated with LV systolic dysfunction, however with a significant improvement of the global LV contractility (Fig. 3a and b), absence of myocardial fibrosis in the late gadolinium enhancement sequences (Fig. 3c), signs of myocardial edema in the LV wall in T2-weighted sequences and moderate pericardial effusion (Fig. 3d), suggestive with Takotsubo cardiomyopathy.

Treatment with carvedilol 3.125 mg twice daily and enalapril 2.5 mg twice daily (titrating the dose up to the maximum dose tolerated by the patient) was initiated. The levels of troponin-I returned to the normal range after 4 days, CKMB levels after 6 days and BNP in 12 days. Fifteen days after the event, the patient presented with chest pain, and pericarditis was diagnosed. Repeat echocardiogram after 12 days of the TTS event, showed remarkable improvement in LV function, with LVEF of 68%, preserved biventricular function and small pericardial effusion. The patient was treated with Ibuprofen 800 mg three times per day and Colchicine 0.5 mg twice daily for three months, with good response and no signs of recurrence. The cardiac MRI was repeated two months later, showing a preserved biventricular contractility, a physiological pericardial effusion, and no late gadolinium enhancement suggestive of myocardial fibrosis. Once complete cardiac recovery was achieved, she was able to resume brachytherapy, and the remaining application was performed 15 days later as an inpatient condition and under rigorous monitoring, uneventfully. The total cancer treatment time was 64 days.

The patient received further adjuvant treatment with cisplatin 50 mg/m^2^ plus gemcitabine 100 mg/m^2^, with discontinuation after one cycle due to severe myelotoxicity. Further, she continued her therapy with carvedilol and enalapril for approximately 6 months when she spontaneously decided to interrupt the treatment. The patient remains in follow-up with sustained complete response from the cancer as demonstrated by pelvic MRI and clinical examination performed every six months for two years until the time that this manuscript was submitted to publication.

## Discussion

This report illustrates the case of a young patient with locally advanced endocervical adenocarcinoma, FIGO stage IIA1, that was recommended to treatment with concurrent chemoradiotherapy and presented TTS followed by cardiorespiratory arrest induced during brachytherapy.

TTS is clinically an acute myocardial infarction-like cardiomyopathy without a culprit coronary artery lesion [3]. Approximately 80–90% of cases occur in postmenopausal women and classically TTS has been linked to emotional stress due to excessive release of catecholamines as mechanism of response [1, 9, 10]. Although the patient in this case outwardly displayed very little anxiety about her diagnosis and treatment approach, it is possible that she felt a heightened degree of internal emotional stress that may have contributed to the development of TTS. It is also possible that she presented some degree of pain during applicator removal at the end of the third brachytherapy session, due to anesthesia superficialization, which could have triggered a cardiomyopathy induced by the release of several inflammatory cytokines and metabolites related to this physical distress. Of note, a small vaginal laceration was observed after the applicator removal.

An important subject of our case is the differential diagnosis with myocarditis. The presence of pericardial effusion is not part of the diagnosis of TTS and it is very common in patients with myocarditis. The absence of late gadolinium enhancement by cardiac RMI may also be present in patients with myocarditis, especially in the initial days. However, the cardiac MRI performed 2 months later to the event did not demonstrate any sign of myocardial fibrosis, which helped to rule out the hypothesis of myocarditis.

The relationship between malignancy and TTS is particularly interesting from an epidemiologic, mechanistic and outcome standpoint as both malignancy and chemotherapy have been associated with TTS [11]. The overall long-term mortality of TTS patients with malignant disease is significantly increased and the prevalence of cancer in patients with TTS is high, considerably exceeding that in the normal population [12]. One of the hypothesis is that cancer and TTS share similar triggering mechanisms, which consist in activation of the sympathetic nervous system [8, 13]. Therefore, TTS patients with cancer should be considered a high-risk subgroup with respect to their increased mortality rate.

Endothelial dysfunction in epicardial and microvascular coronary arteries occurs frequently in patients with cancer, especially during and after systemic chemotherapy or radiotherapy of the heart region, which might be a predisposing factor for the developement of TTS [1, 3, 14] Mediastinal radiotherapy can induce heart disease, such as coronary obstruction, stenosis or regurgitation due to valvular fibrosis, cardiomyopathy and pericardial constriction and inflammation [15]. Though cisplatin remains the cornerstone of endocervical carcinoma stage IIA1 treatment chemotherapy and a standard of care, however it is also related to some other rare side effects like vasculitis, cardiomyopathy and vascular thrombosis. Cisplatin inducing cardiomyopathy and thromboembolic phenomena can be explained by several mechanisms, some of which are endovascular injury with intimal fibrosis, decreased activation of protein C, hypomagnesaemia and myocardial fiber apoptosis [11]. Previous usage of cisplatin might have contributed for the development of myocardial dysfunction in our patient.

Although, so far there is limited data on TTS induced by radiotherapy and no reports on brachytherapy-induced cardiomyopathy. In fact, brachytherapy by itself is painless. However, applicators placement and removal may present only mild discomfort or severe pain according to the different procedures (molds, intracavitary procedures, interstitial implants), and the use of sedation or anesthesia is properly indicated. TTS in the context of intracavitary gynecological brachytherapy procedure, not the radiation delivery, could have been triggered by the unweighted pain experienced by our patient during applicator removal. This case may represent a warning for intracavitary gynecological brachytherapy procedures, since in many facilities in our country it is performed only with sedation, or even without any sedation or analgesia.

Although most patients with TTS experience complete cardiac function recovery, the complication rates can be compared to those found in patients with acute coronary syndrome [13]. Patients who survive an acute episode typically recover systolic function within 1 to 4 weeks [1, 3]. Our patient recovered the systolic function within 2 weeks.

## Conclusions

TTS appears to occur more frequently than was previously thought. Increased awareness of the existence of this syndrome and knowledge of its risk factors are gaining importance in recent years. Further research is required to investigate the interplay of malignancies, its treatments and TTS, which might be helpful to elucidate the pathophysiological mechanism of this cardiomyopathy. To the best of our knowledge, we are herein reporting the second case of TTS arising in the setting of radiation therapy and the first for brachytherapy.

## Data Availability

Not applicable.

## Abbreviations

TTS: Takotsubo syndrome
ECG: Electrocardiographic
LV: Left ventricle
CKMB: creatine kinase-MB
ACS: Acute coronary syndrome
MRI: Magnetic resonance imaging
LVEF: Left ventricular ejection fraction
PET-CT: Positron Emission Tomography – Computed Tomography
